# Bioshell Calcium Oxide (BiSCaO) Ointment for the Disinfection and Healing of *Pseudomonas aeruginosa*-Infected Wounds in Hairless Rats

**DOI:** 10.3390/ijms21114176

**Published:** 2020-06-11

**Authors:** Tomohiro Takayama, Masayuki Ishihara, Shingo Nakamura, Yoko Sato, Sumiyo Hiruma, Koichi Fukuda, Kaoru Murakami, Hidetaka Yokoe

**Affiliations:** 1 Department of Oral and Maxillofacial Surgery, National Defense Medical College, 3-2 Namiki, Tokorozawa, Saitama 359-8513, Japan; murakami@ndmc.ac.jp (K.M.); yokoe@ndmc.ac.jp (H.Y.); 2 Division of Biomedical Engineering, Research Institute, National Defense Medical College, 3-2 Namiki, Tokorozawa, Saitama 359-8513, Japan; ishihara@ndmc.ac.jp (M.I.); snaka@ndmc.ac.jp (S.N.); res337@ndmc.ac.jp (S.H.); khf05707@nifty.com (K.F.); 3 Division of Statistical Analysis, Research Support Center, Shizuoka General Hospital, 4-27-1 Kita-ando, Aoi-ku, Shizuoka 420-8527, Japan; sato.yoko.shiz@gmail.com

**Keywords:** bioshell calcium oxide (BiSCaO), bactericidal activity, ointment, infected wound, wound repair

## Abstract

Bioshell calcium oxide (BiSCaO) possesses deodorizing properties and broad microbicidal activity. This study aimed to investigate the application of BiSCaO ointment for the prevention and treatment of infection in chronic wounds in healing-impaired patients, without delaying wound healing. The bactericidal activities of 0.04, 0.2, 1, and 5 wt% BiSCaO ointment, 3 wt% povidone iodine ointment, and control (ointment only) were compared to evaluate the in vivo disinfection and healing of *Pseudomonas aeruginosa*-infected wounds in hairless rats. Treatment of the infected wounds with 0.2 wt% BiSCaO ointment daily for 3 days significantly enhanced wound healing and reduced the in vivo bacterial counts compared with povidone iodine ointment and control (no wound cleaning). Although 5 wt% BiSCaO ointment provided the lowest bacterial counts during 3 days’ treatment, it delayed wound healing. Histological examinations showed significantly advanced granulation tissue and capillary formation in wounds treated with 0.2 wt% BiSCaO ointment for 3 days compared to wounds treated with the other ointments. This study suggested that using 0.2 wt% BiSCaO ointment as a disinfectant for infected wounds and limiting disinfection to 3 days may be sufficient to avoid the negative effects of BiSCaO on wound repair.

## 1. Introduction

Wound healing is the result of a series of correlated cellular processes that are initiated by cytokines and growth factors [1]. These cellular processes are suppressed by tissue bacterial bioburden [2], which may contribute to degradation of the cytokines and growth factors [3]. Several studies have shown that the level of bacterial bioburden can exceed 1 × 10^6^ per gram of tissue [4]. Such high levels of tissue bacteria can be present without clinical signs of infection and can unfavorably affect wound healing [5]. 

*Pseudomonas (P.) aeruginosa* is a major nosocomial microbe and opportunistic pathogen that can infect wounds and is known to play a role in impaired wound healing [6]. In a chronic granulating wound, systemically administered antibiotics do not effectively decrease the level of bacteria [6]. Furthermore, the topical use of antibiotics used systemically for purposes other than wound infection is discouraged because of an increased risk for allergies and the potential for drug resistance [7]. Antiseptics and non-antibiotic antimicrobials, such as povidone iodine and weakly acidic hypochlorous acid (HClO) solution, have been demonstrated to be cytotoxic to the cellular components of wound healing [8,9,10]. Therefore, a topical antimicrobial that can decrease the bacterial bioburden in chronic wounds without inhibiting the wound-healing process is a therapeutic imperative [6].

Calcium oxide (CaO) is generally produced from limestone and is an important inorganic compound used in various industries as an adsorbent for toxic waste remediation and as an alkalization agent. However, CaO derived from limestone contains harmful impurities and rapidly generates heat upon hydration [11,12]. Scallop shells are an alternative readily available source of CaO that is used as a food additive, as well as in plastering and paving materials. However, CaO derived from scallop shells also contains harmful impurities such as heavy metals and thus most scallop shells are treated as industrial waste and piled on the shores of harvesting districts in Japan, causing serious problems such as offensive odors and soil pollution [13]. 

Heated scallop shell powder (SSP) exhibits strong microbicidal activities [14]. SSP heated at >1000 °C, then ground further, exhibits broad microbicidal action against various viruses, bacteria, heat-resistant bacterial spores, fungi and biofilms [12,13,14,15,16,17,18,19], and is used as an additive to prolong the shelf life of food [14,19].

Slurries of SSP comprising particles with diameters in the range of 60–900 nm are prepared by grinding shells heated at >1100 °C with a wet bead grinding mill [14] and suspending the powder in sterile saline. The main component of this heated shell powder slurry is calcium hydroxide (Ca(OH)_2_). Similarly, most commercially available heated shell powder products used as food additives comprise Ca(OH)_2_. In a previous study, we used commercially available scallop shell powders with an average diameter of 6 μm heated at 1450 °C (BiSCaO) purchased from Plus Lab Corp., Kanagawa, Japan. According to the manufacturer, the content of CaO exceeded 99.5% [20,21].

Several techniques have been developed for cleaning chronic infected wounds, leg ulcers and pressure ulcers using water or saline at pH values of between 5.5 and 6.5 and at temperatures of between 35 °C and 45 °C [22,23,24]. Weakly acidic (pH 5.5–6.5) hypochlorous acid (HClO) solution generated by mixing sodium hypochlorite and weakly acidic water or saline has excellent In Vitro bactericidal properties [25,26]. Daily wound cleaning with HClO solution (pH 6.5) for 12 days decreased the *P. aeruginosa* bioburden in infected wounds in db/db diabetic mice but wound repair was delayed [27]. 

Limiting disinfection treatment with both HClO and chitin-nanofiber sheet-immobilized silver nanoparticles (CNFS/Ag NPs) to 3 days [28,29,30] may suppress these negative effects on wound repair [31]. We previously showed that treating *P. aeruginosa*-infected wounds on hairless rats with BiSCaO suspension (0.2 wt%, pH 12.3) once daily for 3 days and covering the wound with CNFS significantly decreased the *P. aeruginosa* bioburden and enhanced wound repair [32]. 

The aim of the present study was to test the originally proposed various concentrations of BiSCaO in an ointment rather than in a slurry, with the eventual aim of preventing and treating infection in chronic wounds in healing-impaired patients, without delaying wound healing.

## 2. Results

### 2.1. Bactericidal Activities of Various Concentrations of BiSCaO and Povidone Iodine Ointments In Vitro

**Figure 1:**
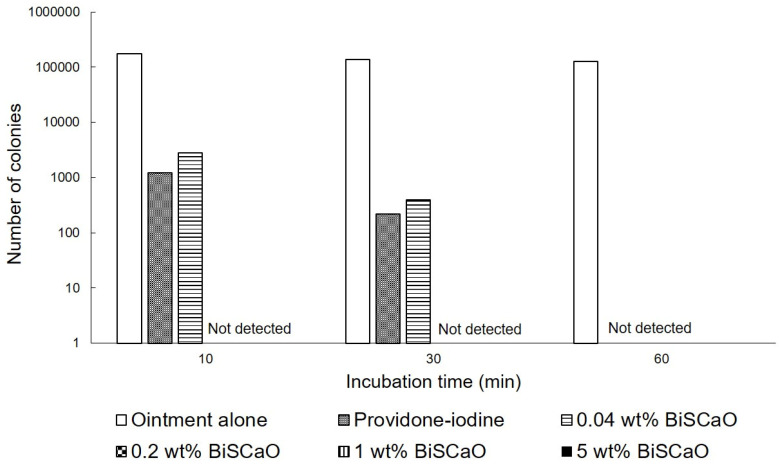
In vitro bactericidal activity of each ointment. *P. aeruginosa* was exposed to 0.04, 0.2, 1 and 5 wt% BiSCaO ointments, 3 wt% providone iodine ointment, or control ointment-coated plates and incubated for 5, 10, 20 and 30 min at room temperature. The bacterial colonies were counted to measure the minimal bactericidal concentration of each disinfectant (*n* = 6).

The in vitro bactericidal activities of different concentrations of BiSCaO ointments and 3 wt% povidone iodine ointments against *P. aeruginosa* were tested by counting the viable bacterial colonies after treatment. The colonies of *P. aeruginosa* were completely eradicated after exposure to more than 0.2 wt% BiSCaO after 10 min, but some colonies survived after exposure to 0.04 wt% BiSCaO and 3 wt% providone iodine ointments (Shionogi Co., Ltd., Osaka, Japan) after 10 and 30 min. All 0.04, 0.2, 1 and 5 wt% BiSCaO ointments, and 3 wt% providone iodine ointment, completely eradicated the colonies after 60 min whereas control ointment alone did not decrease *P. aeruginosa* after 1 h (Figure 1).

### 2.2. Disinfection of P. aeruginosa-Infected Wounds with BiSCaO and Povidone Iodine Ointments In Vivo

All animals tolerated the creation of *P. aeruginosa*-infected wounds, and daily wound cleaning with saline and application of ointment without complications. No signs of acute inflammation, abscess formation, or seroma accumulation were seen on the infected wound sites on the 9th day after surgery and infection.

**Figure 2:**
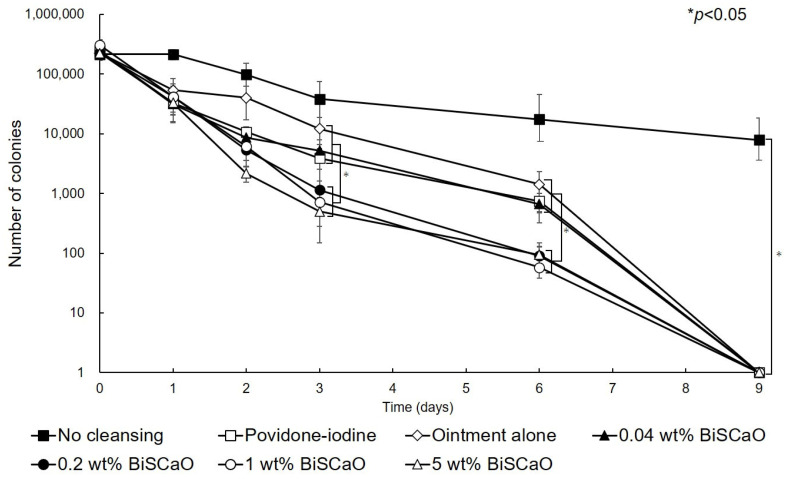
Eradication of *P. aeruginosa* colonies from wounds treated with disinfectant In Vivo. The *P. aeruginosa*–infected wounds on hairless rats were cleaned with saline, then each ointment was applied once daily on Days 1–3 and covered with chitin nanofiber sheets (CNFS). Wounds cleaned with saline once daily on Days 4–9 were covered with CNFS. After cleaning with saline, the contents of the wounds on the indicated day after wound creation were collected and the viable cell counts were measured. Data are presented as mean ± standard deviation (SD, *n* = 6).

Before cleaning the wound and applying each ointment (Day 0), 2.5 ± 0.4 (×10^5^) colony forming units (CFU) of *P. aeruginosa* were inoculated into each wound. On Day 1 after treating the wounds with 0.04, 0.2, 1.0 or 5.0 wt% BiSCaO, 3 wt% providone iodine-ointment, or control ointment, the mean viable cell counts were 3.14 × 10^4^, 4.18 × 10^4^, 4.15 × 10^4^, 3.3 × 10^4^, 3.24 × 10^4^, and 5.4 × 10^4^, respectively, and are not significantly different. The mean viable cell count in non-cleaned wounds was 2.6 × 10^5^ CFU, which is significantly higher than the other groups. These results show that the bioburden was effectively decreased by cleaning with saline and treating with BiSCaO immediately following cleaning whereas the bioburden slightly increased in the non-cleaned wound group. On Day 2, the mean viable cell count of the 5 wt% BiSCaO ointment group was lower than the other groups. On Days 3 and 6, the mean viable cell counts of the 0.2, 1.0 and 5 wt% BiSCaO ointment groups were significantly lower than those of the 0.04 wt% BiSCaO-ointment, povidone iodine ointment, control ointment, and non-cleaned wound groups. On Day 9, all the *P. aeruginosa* colonies were eradicated in all but the non-cleaned wound group, which had a mean viable cell count of 7.8 × 10^3^ (Figure 2). These results suggested that cleaning the infected wounds with saline and application of 0.2, 1 or 5 wt% BiSCaO ointment for 3 days significantly decreased the mean viable cell counts on Days 3 and 6 compared to the other groups, and there were no significant differences between the three concentrations of BiSCaO.

### 2.3. Healing of P. aeruginosa-Infected Wounds by Applying Disinfectants In Vivo

**Figure 3:**
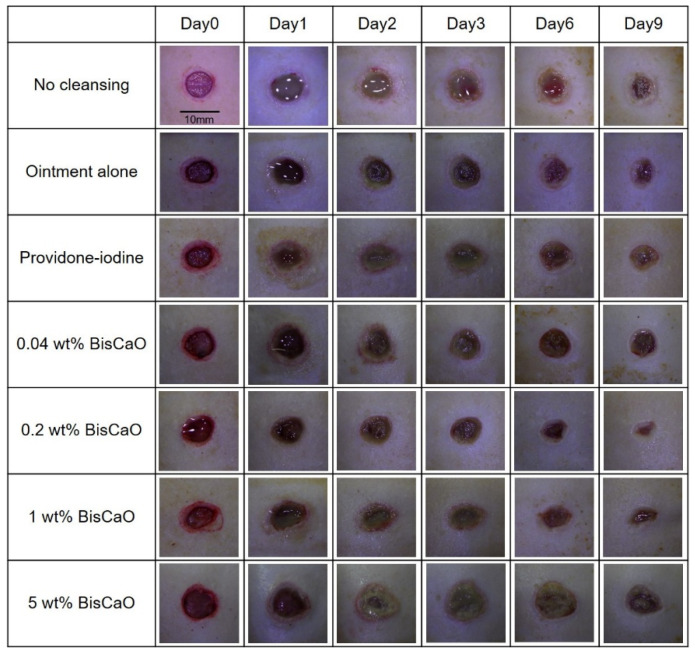
Digital photographs of the open areas of infected wounds. The *P. aeruginosa*-infected wounds on hairless rats were cleaned with saline, then each ointment was applied once daily on Days 1–3 and covered with CNFS. Wounds cleaned with saline once daily on Days 4–9 were covered with CNFS. The wounds were photographed before cleaning on Days 0, 1, 2, 3, 6 and 9 to allow assessment of the rate of wound healing. The images represent wounds (*n* = 6) of the indicated treatment group on the indicated day.

**Figure 4:**
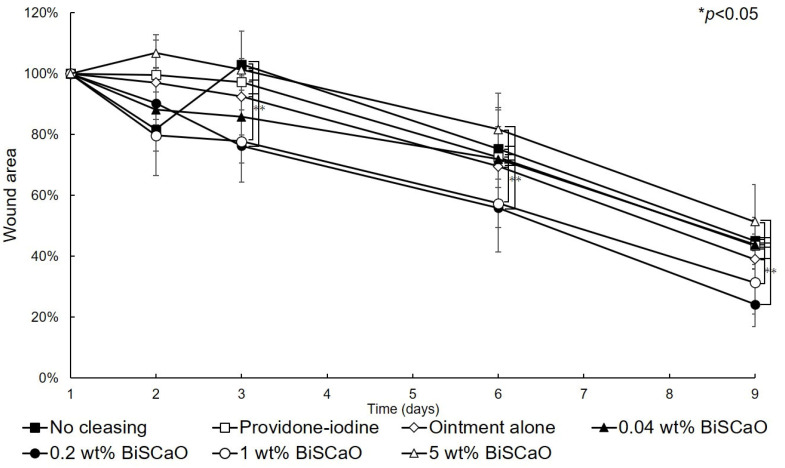
Rate of healing of open wounds. The area of the open wounds on Day 1 was defined as 100%, and the relative open wound area of each group was calculated using the digital photographs in Figure 3. Data are presented as mean ± SD (*n* = 6). * *p* < 0.05; Tukey’s *t*–test.

The open wound areas for all groups was defined as 100% on Day 0 based on digital photographs (Figure 3) and the change in open wound area was monitored with time (Figure 4). There were no significant differences in wound closure rates on Day 2 between the groups (Figure 3 and Figure 4). On Day 3, the open wound areas of the 0.2 wt% BiSCaO ointment (76%) and 1 wt% BiSCaO ointment (78%) groups were significantly smaller than those of the providone iodine ointment (97%), 5 wt% BiSCaO ointment (101%), control ointment (94%) and non-cleaned groups (103%). On Days 6 and 9, the open wounds of the 0.2 and 1 wt% BiSCaO ointment groups were significantly smaller than those of the other groups. Although the result did not indicate wound contraction, which is an important factor for acceleration of wound healing [33,34,35], BiSCaO ointment might induce wound contraction in addition to disinfection for *P. aeruginosa*. These results suggested that application of 0.2 and 1 wt% BiSCaO ointment for 3 days may help eradicate *P. aeruginosa* and aid wound repair.

### 2.4. Histological Analyses

**Figure 5:**
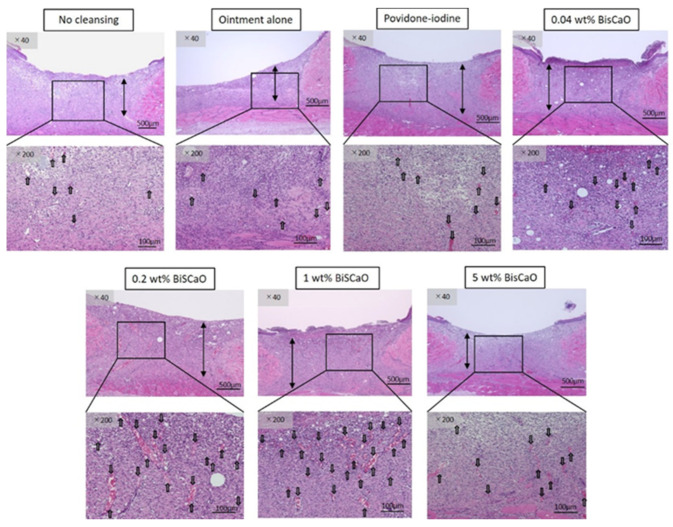
Histological examination of granulation tissue and capillary formation in each treatment group. The skin surrounding the infected wounds in each treatment group on Day 9 was harvested, processed for hematoxylin–eosin (H&E) staining, and microphotographed (*n* = 6) (magnification: 40×, 200×), the arrows show blood vessels.

**Figure 6:**
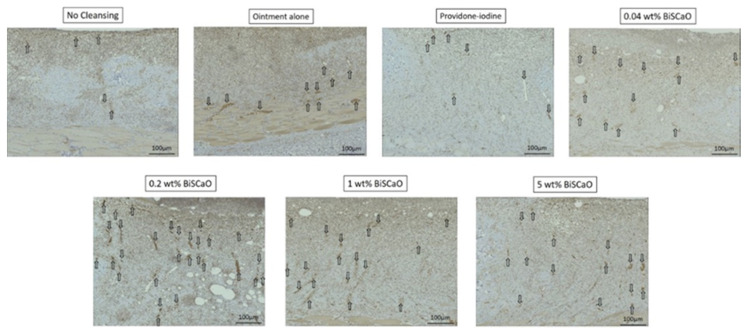
Histological examination of capillary formation with CD-34-stained immunophotographs in each treatment group. The skin surrounding the infected wounds in each treatment group on Day 9 was harvested, processed for CD 34-staining, and microphotographed (*n* = 6) (magnification: 200×). The arrows show blood vessels. The microphotographs represent the wounds for the indicated treatment group. Analysis of these microphotographs provides confirmation of the data given in Table 1.

Histological examinations on Day 9 showed that granulation tissue formation and vascularization (Figure 5) in the 0.2 and 1 wt% BiSCaO ointment groups were significantly higher than those of the other groups (Table 1). Furthermore, CD-34-stained immunophotographs (Figure 6) confirmed capillary densities of each groups.

The bars show generated granulation tissues and the arrows show blood vessels. The microphotographs represent the wounds for the indicated treatment group. Analysis of these microphotographs provides the data given in Table 1.

## 3. Discussion

The infection of wounds with *P. aeruginosa* is a major complication in clinical settings. In this study, in vitro bactericidal tests against *P. aeruginosa* showed complete eradication within 15 min of treatment with 0.2, 1 and 5 wt% BiSCaO ointments but 60 min with 0.04 wt% BiSCaO and 3 wt% povidone iodine ointments (Figure 1). We evaluated bactericidal activity and wound healing in vivo by cleaning *P. aeruginosa*-infected wounds on hairless rats with saline, applying each ointment, and covering with CNFS for 3 days. Each subsequent day for 6 days, the wounds were cleaned with saline, each ointment was applied, and the wound was covered with CNFS. The results suggested that applying 0.2–1 wt% BiSCaO ointment for 3 days to the infected wounds significantly enhanced disinfection and wound healing in vivo, compared with the effects of povidone iodine ointment and control ointment. Histological examination showed significantly advanced formation of granulation tissue and capillaries following application of 0.2–1 wt% BiSCaO to wounds for 3 days. Our previous study on FGF-containing photocrosslinkable chitosan hydrogel as wound dressing showed that wound contraction was an important factor for acceleration of wound healing [33,34,35]. It is possible BiSCaO ointment might induce wound contraction in addition to disinfection for *P. aeruginosa*, although we do not have direct data on the effect of BiSCaO on contraction and/or epithelialization. Furthermore, treatment with each ointment for 3 days did not produce signs of complications, as confirmed by histological analysis of wound skin harvested on Day 9. These results suggested that limiting disinfection by BiSCaO treatment to 3 days may be appropriate in a clinical situation, such as for the prevention and treatment of infection of chronic wounds in healing-impaired patients. We suggest that 0.2 wt% BiSCaO ointment is optimal in terms of cost-effectiveness and safety.

Systemically administered antibiotics do not effectively decrease the level of bacteria in a chronic granulating wound because of an increased risk of developing allergies and the potential for selection of bacteria resistant to the drug [6,7] Antiseptics and non-antibiotic antimicrobials such as povidone-iodine, silver sulfadiazine, silver nanoparticles, mafenide acetate cream, and weakly acidic hypochlorous acid (HClO) solution have been demonstrated to be cytotoxic to the cellular components of wound healing. On the other hand, hydrogel dressings with antioxidant functions have emerged and are proven to accelerate wound healing, especially for chronic wound repair [36,37].

Clinical antiseptics such as povidone iodine and HClO are cytotoxic to the cellular components of wound healing and high concentrations are required for disinfection [7,8,9]. Therefore, a topical disinfectant that can decrease the bacterial bioburden of chronic wounds without inhibiting the wound-healing process is a therapeutic imperative. Although both BiSCaO and commercially available heated SSP-Ca(OH)_2_ are poorly water-soluble at strong alkaline pH (pH ≧ 12), BiSCaO and SSP-Ca(OH)_2_ suspensions in water generate a strong base, which is the primary mechanism for their microbicidal activities. The high disinfection activity of suspensions of BiSCaO and SSP-Ca(OH)_2_ particles might be due to the higher OH^–^ concentration of the thin water layer surrounding the particles compared to the bulk solvent [38,39]. However, we found that the disinfection activity of BiSCaO for both coliform bacteria (CF) and total viable cells (TC) was higher than that of SSP-Ca(OH)_2_ at identical pH [38,39], suggesting that the high disinfection activity of BiSCaO is due to a different mechanism. Several researchers reported that heated shell powders comprising mainly CaO had higher disinfection activity than those composed of mainly Ca(OH)_2_ for the deactivation and removal of biofilms of *Escherichia coli* [40] and *Listeria* species [19]. We hypothesized that a higher concentration of OH^–^ in the thin aqueous surface layer of BisCaO particles might damage and kill various bacterial cells when BiSCaO particles come in contact with contaminated skin wound surfaces. Furthermore, heated SSP composed mainly of CaO but lacking Ca(OH)_2_ (i.e., BiSCaO) was previously reported to generate reactive hydroxyl radical species, which may contribute to the stronger disinfection activity heated SSP compared to SSP-Ca(OH)_2_ [19,40]. Our preliminary experiment also confirmed that BiSCaO also generate hydroxyl radical species. Low levels of reactive oxygen species are conductive to normal wound healing, but excessive reactive oxygen species can hinder wound healing [36]. Therefore, concentration of BiSCaO ointment (0.2–1 wt%) and time period (3 days) for the treatment should be limited to avoid the negative effects of BiSCaO on wound repair, as shown in this study. Thus, limited application of 0.2 wt% BiSCaO ointment, not 5 wt%, to the infected wounds for 3 days optimally enhanced both disinfection and wound healing.

Compared with basic NaClO solution, low concentrations of weakly acidic HClO solution (50–200 ppm) are reported to have excellent In Vitro bactericidal properties against Gram-positive organisms such as *Staphylococcus aureus, Bacillus cereus* and *Bacillus subtilis,* and Gram-negative bacteria such as *P. aeruginosa* [41,42]. However, weakly acidic HClO reacts readily with various NH_2_- or CHO-containing organic compounds (e.g., proteins, amino acids and carbohydrates), which can rapidly consume HClO in the vicinity of the infected wound [25,43]. A study demonstrated that HClO interacts with primary amino groups (–NH_2_) in organic compounds such as amino acids, generating chloramine groups (–NH_2_Cl or –NHCl_2_) that are known to have oxidizing properties and antimicrobial activity [44]. 

BiSCaO has not been approved by the Pharmaceuticals and Medical Devices Agency of Japan for use as a pharmaceutical or medical device, despite the approval of NaClO for such purposes. Additional systemic studies on BiSCaO are required to establish its efficacy, safety, and stability for medical use. Furthermore, since we have preliminary data which 0.2 wt% BiSCaO suspension has bactericidal activity against methicillin-resistant *Staphylococcus aureus* (MRSA), the effects on MRSA would be investigated to apply this BiSCaO ointment for clinical practice.

## 4. Materials and Methods

### 4.1. Preparation of BiSCaO Ointment

**Figure 7:**
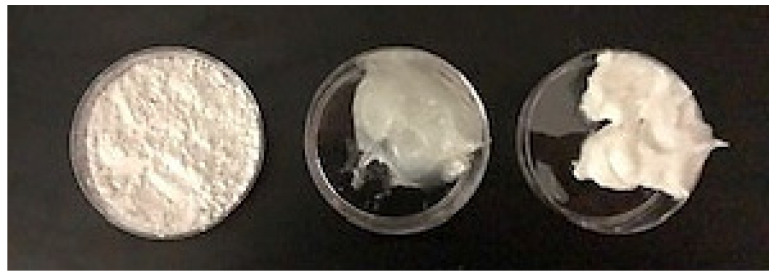
Appearance of BiSCaO (**left**), ointment (white Vaseline, middle) and 0.2 wt% BiSCaO ointment (**right**).

Scallop shell powders with an average diameter of 6 μm heated at 1450 °C (BiSCaO) were purchased from Plus Lab Corp., Kanagawa, Japan. According to the manufacturer, the content of CaO exceeded 99.5%. BiSCaO ointments (5, 1, 0.2 and 0.04 wt%) were prepared by mixing well BiSCaO with white Vaseline (KENEI Pharmaceutical Co., Ltd., Osaka, Japan). (Figure 7). Povidone iodine ointment (3 wt% ISODINE^®^) was purchased from Shionogi Co., Ltd., Osaka, Japan.

### 4.2. Bactericidal Activity of Various Concentrations of BiSCaO and Povidone Iodine Ointments In Vitro

*P. aeruginosa* (American Type Culture Collection 27853, Manassas, VA, USA) colonies were stored at −80 °C in Luria–Bertani broth containing 50% sterile glycerol and were freshly grown at a density of 1.0 × 10^6^ colony forming units (CFU)/mL. Various concentrations of BiSCaO ointment, providone iodine ointment, and control ointment (1 mL) were coated onto 90 × 15 mm Petri plates. *P. aeruginosa* suspension (5 mL) was added to each ointment-coated plate and incubated for 10, 30 and 60 min at room temperature. Each *P. aeruginosa* suspension was plated onto 90 × 15 mm Petri plates containing *Pseudomonas* isolation agar (Neogen Ltd., Lansing, MA, USA) and incubated at 37 °C for 24 h. The generated colonies were counted and disinfection activity was evaluated to determine in vitro bactericidal activity against *P. aeruginosa*.

### 4.3. Applications of Various Concentrations of BiSCaO and Povidone Iodine Ointment to P. aeruginosa-Infected Wounds In Vivo

All animal experiments were approved by the National Defense Medical College, Tokorozawa, Saitama, Japan, and were carried out following the relevant guidelines for animal experimentation (approval number, 17084, 18/2/2019). Hairless rats (male, 300–350 g) were obtained from Japan SLC Inc., Shizuoka, Japan and were maintained under appropriate conditions (i.e., 26 °C, 55% humidity). On nominal Day 0 of the study, the rats were placed under general anesthesia by intraperitoneal injection of pentobarbital sodium (Dainippon Sumitomo Pharma Co., Ltd., Osaka, Japan). Full-thickness round wounds were created on the back of each rat using a sterile 8 mm dermal punch (Kai Industries Co., Ltd., Oyana, Japan) and a pair of sterilized sharp scissors. To generate infected wounds, 100 µL of growing *P. aeruginosa* was applied to the surface of each freshly generated wound, and each wound was covered with a piece of chitin nanofiber sheet (CNFS) approximately 30% deacetylated and obtained as a commercial product (BeschitinW, Nipro Corp., Osaka, Japan). The animals were returned to their cages and the wounds were visibly infected 24 h later. The *P. aeruginosa*–infected wounds were cleaned once daily by gentle rubbing 3 times using gauze dipped in 3 mL of saline (Ootsuka normal saline, Otsuka Pharmaceutical Factory, Inc. Tokushima, Japan), then 1 mL of 0.04 wt%, 0.2 wt%, 1 wt% or 5 wt% BiSCaO ointment, 3 wt% providone iodine ointments, or control ointment was applied, then covered with CNFS for the first 3 days. On Days 4–9, all wounds in the 4 groups were cleaned with saline and covered with CNFS daily. The infected wounds in the non-cleaned group were only covered with CNFS without cleaning for the 9 days of the experimental period. After cleaning the wounds on Days 1, 2, 3, 6 and 9, the bioburden of each infected wound was determined by wiping with a strip of sterile 1 cm^2^ gauze. In the untreated group, the viable cells were counted after removal of the CNFS. The resulting cell suspensions were serially 10-fold diluted and 100 µL aliquots of the diluted suspensions were plated onto 90 mm × 15 mm Petri plates containing *Pseudomonas* isolation agar. The plates were incubated at 37 °C for 24 h; the viable cells confirmed to be *P. aeruginosa* by observation of their morphology were counted [26]. Digital photographs were recorded on Days 0, 1, 2, 3, 6 and 9 and used to measure the rate of wound closure and to confirm the absence of complications, including acute inflammation, abscess formation, and seroma accumulation. 

### 4.4. Histological Examination

Following the post-cleaning collection of wound contents on Day 9, the animals were euthanized using pentobarbital sodium and the skin surrounding each infected wound, including wound tissue, was removed from each rat (*n* = 6) for histological examination. Skin samples from each treatment group were fixed in 10% formaldehyde solution, embedded in paraffin, sectioned in 4 µm increments (Yamato Kohki Inc., Asaka, Saitama, Japan) and used to generate 10 × 1.5 mm sections perpendicular to the anterior–posterior axis and to the surface of the wound. These sections were transferred to glass slides for staining with hematoxylin–eosin (H&E) reagent and for CD-34 immunostaining, covered with a cover slip, and evaluated microscopically. In each section (*n* = 8), the microscopic field showing the wound was photographed and the number of capillary lumens ≥ 10 µm in diameter or containing ≥ 5 erythrocytes was counted. 

### 4.5. Statistical Analyses

Results were expressed as mean ± standard deviations (SDs). Tukey’s test was used to compare the disinfecting solutions. The statistical software JMP^®^ (SAS Institute Inc., Tokyo, Japan) was used for the analyses. A value of *p* < 0.05 was considered to be statistically significant.

## 5. Conclusions

In conclusion, treatment of *P. aeruginosa*-infected wounds on hairless rats by cleaning once a day with saline, applying 0.2 wt% BiSCaO ointment for 3 days, and covering with CNFS significantly decreased the *P. aeruginosa* bioburden and enhanced wound repair. 

## Figures and Tables

**Table 1:** Histological examination of the length of granulation tissue and capillary formation.

Disinfectant in Ointments	Length of Granulation Tissue (µm)	Capillary Formation (Capillary Number/Image)
No cleansing	920 ± 95	7 ± 5
Ointment alone	940 ± 100	13 ± 4
Providone-iodine	980 ± 80	9 ± 3
0.04 wt% BiSCaO	1020 ± 60	14 ± 6
0.2 wt% BiSCaO	1160 ± 85 *	35 ± 10 *
1 wt% BiSCaO	1120 ± 75 *	31 ± 8 *
5 wt% BiSCaO	950 ± 65	11 ± 6

The data represent the mean ± SD. In length of granulation tissue, * *p* < 0.05 vs. no cleansing and ointment alone (*n* = 6), in capillary formation, * *p* < 0.05 vs. no cleansing and providone-iodine (*n* = 6).

## Abbreviations

BiSCaO	bioshell calcium oxides
SSP	heated scallop shell powder
CNFS	chitin nanofiber sheets
Ag NP	silver nanoparticles
HClO	hypochlorous acid
CFU	colony forming units
